# Atherosclerotic ICA stenosis coinciding with ICA asymmetry associated with Circle of Willis variations can mimic near-occlusion

**DOI:** 10.1007/s00234-019-02309-7

**Published:** 2019-11-08

**Authors:** Elias Johansson, Richard I. Aviv, Allan J. Fox

**Affiliations:** 1 Department Clinical Science, and Wallenberg Center for Molecular Medicine (WCMM), Umeå University, Umeå, Sweden; 2 Department of Medical Imaging, Sunnybrook Health Science Center, University of Toronto, Toronto, Canada

**Keywords:** Computed tomography angiography, Carotid artery, Carotid stenosis, Near-occlusion

## Abstract

Differentiating carotid near-occlusion (tight atherosclerotic stenosis causing distal artery size reduction) from conventional stenosis is the first step when grading carotid stenoses with NASCET method. The internal carotid artery (ICA) can be asymmetrically associated with Circle of Willis variations. When such ICA asymmetry coincides with stenosis, it may mimic near-occlusion. We studied ICA anatomical variant prevalence in 4042 consecutive CTA exams from all indications, 53 excluded due to carotid occlusion, 814 with any ≥ 50% steno-occlusive disease intra- or extracranially, 3228 without. Of the 3989 included cases, 568 (14%) had ICA asymmetry, of which 335 (59%) were from associated with Circle of Willis variations. Of 3228 patients without ≥ 50% stenosis or other steno-occlusive disease intra- and extracranially; 257 (8.0%) demonstrated ICA asymmetry associated with Circle of Willis variations, equally common among sexes and age unrelated and most frequently attributed to an ipsilateral A1 hypoplasia/aplasia, less often attributed to large contralateral posterior communicating artery. As ICA asymmetry associated with Circle of Willis variations are common, caution should be exercised diagnosing near-occlusion on asymmetry alone.

## Introduction

When grading carotid stenosis with NASCET-method, the first step is to exclude near-occlusion with or without full collapse by determining that there is distal internal carotid artery (ICA) luminal reduction caused by proximal ICA stenosis [1–3].

**Fig. 1:**
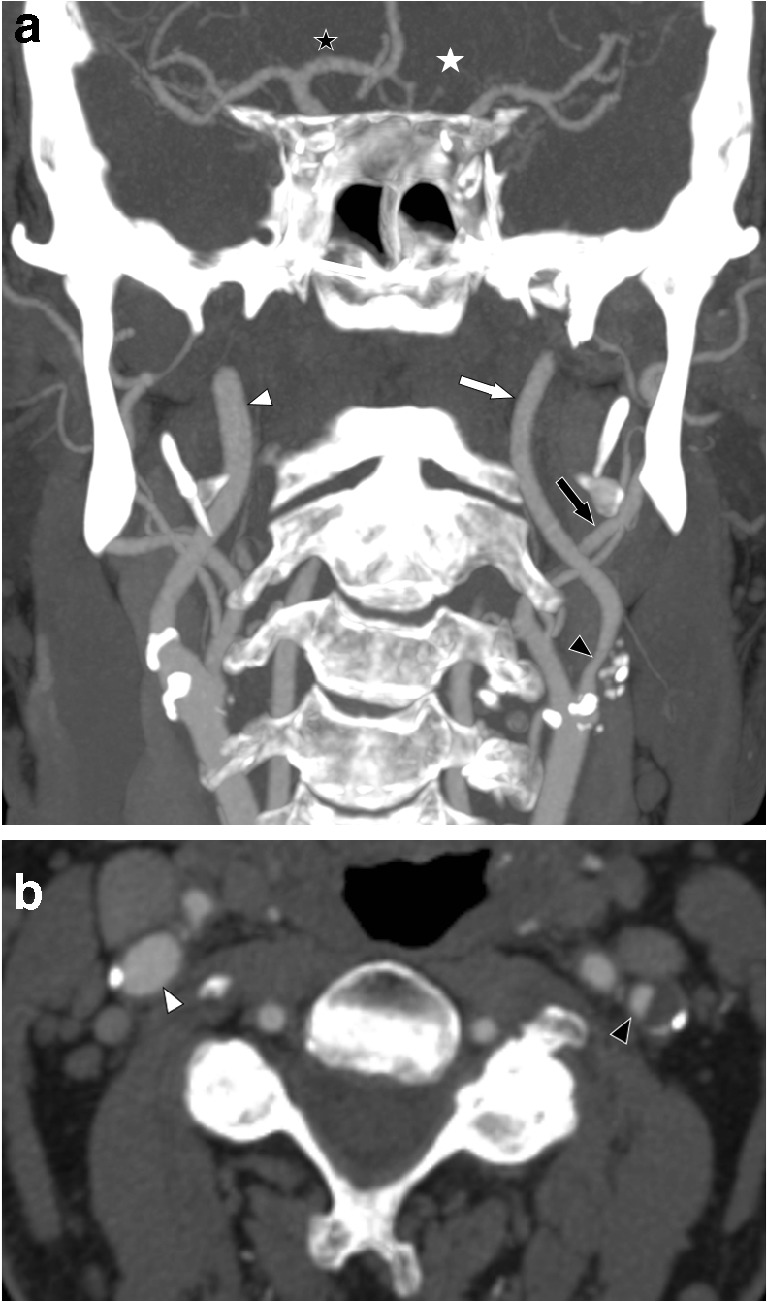
ICA anatomical variance coinciding with a carotid stenosis. **a** Coronal view. Clearly visible cervical ICA asymmetry. Right ICA diameter 5.4 mm (white arrowhead). Left ICA diameter 3.6 mm (white arrow). There is left-sided anterior cerebral artery trunk aplasia (white star), whereas right anterior cerebral artery trunk is unusually prominent, in range of middle cerebral trunk which is usually much larger as it now carries the supply to both medial hemispheres especially frontal lobes (black star with white rim). Each ICA size then reflects the amount of blood carried by each, right much larger than left. Although left ICA and left ECA (3.1 mm, black arrow) are quite similar in size, this can now be dismissed as anatomical variance, not near occlusion. Moreover, the left ICA stenosis is not so severe (1.5 mm, black arrowhead). **b** Axial view of the left-sided stenosis (black arrowhead); this stenosis does not calculate as severe enough stenosis (via NASCET methodology) to qualify as severe enough to cause partial ICA collapse. No stenosis on right side (white arrowhead)

In prognostic and management studies, separation of near-occlusion and conventional ≥ 50% carotid stenoses is made by assessing several features of near-occlusion [3–5]. Koskinen et al. recently suggested that a ≥ 1.0-mm side-to-side difference in ICA diameter could be used to separate near-occlusion and conventional ≥ 50% stenosis [6]. However, asymmetry in the ICA caused by Circle of Willis (CoW) variation can also be associated with a 1.0-mm side-to-side difference of extracranial ICA diameter and mimic near-occlusion when coinciding with stenosis (Fig. 1) [7]. ICA asymmetry associated with CoW anatomical variations (when resulting in differences in the ICAs tissue perfusion volume) was understood to be a near-occlusion mimic (especially near-occlusion without full collapse) in previous studies but was not well described [3–5, 7, 8]. The aim of this study was to systematically study asymmetry in the ICA with focus on ICA asymmetry associated with CoW variations as a near-occlusion mimic.

## Materials and methods

One observer (EJ) assessed a 5-year consecutive sample of 4402 CTA exams from 4042 consecutive patients aged ≥ 18 years sent to or performed at the University Hospital of Northern Sweden, Umeå, Sweden. CTA protocols varied across 12 sites, reflecting clinical practice. Cases with extracranial carotid occlusion were excluded. A blinded second observer (AF) assessed all cases with stenosis requiring separation between near-occlusion and ICA anatomical variants. Disagreements were settled by consensus. The most representative exam was used for cases with several exams. Demographic data obtained was age and sex. Artery diameter measurements (using caliper with 0.1-mm steps) and CoW evaluations were performed for all cases with any ICA asymmetry or ≥ 50% carotid stenosis and randomly selected controls with ICA symmetry and < 50% carotid stenosis. The study was approved by the ethical review board in Umeå, Sweden, with the need for informed consent waived due to the observational nature of the study.

ICA asymmetry was defined as a visible side-to-side difference in extracranial ICA diameter well beyond the bulb (approximately at the C2 vertebra level) caused by a pathology or anatomical variance proximal and/or distal to this artery segment. Causes of ICA asymmetry were categorized as the following: near-occlusion, distal disease (distal stenosis or occlusion), ICA hypoplasia (small ICA with a narrow bone canal), CoW anatomical variance and uncertain. CoW anatomical variance co-existing with moderate carotid stenosis was separated from near-occlusion by systematically interpreting several features of near-occlusion: ICA size, stenosis severity, and ICA/external carotid artery ratio [1, 8]. Approximate thresholds, subordinated expert interpretation, have been suggested elsewhere: ICA ≤ 3.3 mm, stenosis ≤ 1.3 mm, and ICA/external carotid artery ratio ≤ 1.27 [8]. “Uncertain” cause of ICA asymmetry was either CoW anatomical variant with coinciding stenosis on the smaller side or near-occlusion, but which could not be determined with sufficient certainty. A1 segments were considered small when visibly smaller than the other A1. Both posterior communicating arteries (Pcom) were graded on an arbitrary 5-point scale: Absent (1p), Pcom<P1 (2p), Pcom≈P1 (3p), Pcom>P1 (4p), and fetal PCA (5p). A Pcom with ≥ 2 points higher than the other Pcom was considered large.

The side with the smaller ICA was considered the index side. We used 95% confidence intervals (95%CI), mean, standard deviation (±), *t* test, and *χ*^2^ test using SPSS 24.0.

## Results

A total of 3989 cases without extracranial occlusion were included, 53 occlusions excluded. There were 568 cases with asymmetric ICAs, associated with CoW anatomical variants (59%, *n* = 335), near-occlusion (27%, *n* = 155), distal disease (7%, *n* = 39), ICA hypoplasia (1%, *n* = 5), and uncertain (6%, *n* = 34).

Of 635 cases with ≥ 50% extracranial carotid stenosis, 385 (61%) had conventional stenosis and symmetric ICAs, 78 (12%) conventional stenosis and ICA asymmetry associated with CoW anatomical variants (61 smaller ICA ipsi- and 17 contralateral to stenosis), 155 (24%) near-occlusion, and 34 (5%) ICA asymmetry of uncertain cause.

Of 3228 cases without any ≥ 50% steno-occlusive disease, 257 (8.0%, 95%CI 7.0–8.9%) had asymmetric ICA associated with CoW anatomical variants. There were no sex (8.6% in women and 7.2% in men, *p* = 0.17) or mean age differences (64 ± 15 years with ICA anatomical variants and 62 ± 16 years without; *n* = 2971, *p* = 0.06). When grouped by decade, ICA anatomical variants were similarly common in all groups (range 6.3–10.8%). In these 257 asymmetric cases, mean smallest ICA diameter was 3.6 ± 0.5 mm, compared to 4.1 ± 0.5 mm in controls (*p* < 0.001). An ICA ratio ≤ 0.88 was 98% sensitive and 99% specific for separating the 257 asymmetric and 257 control cases.

**Fig. 2:**
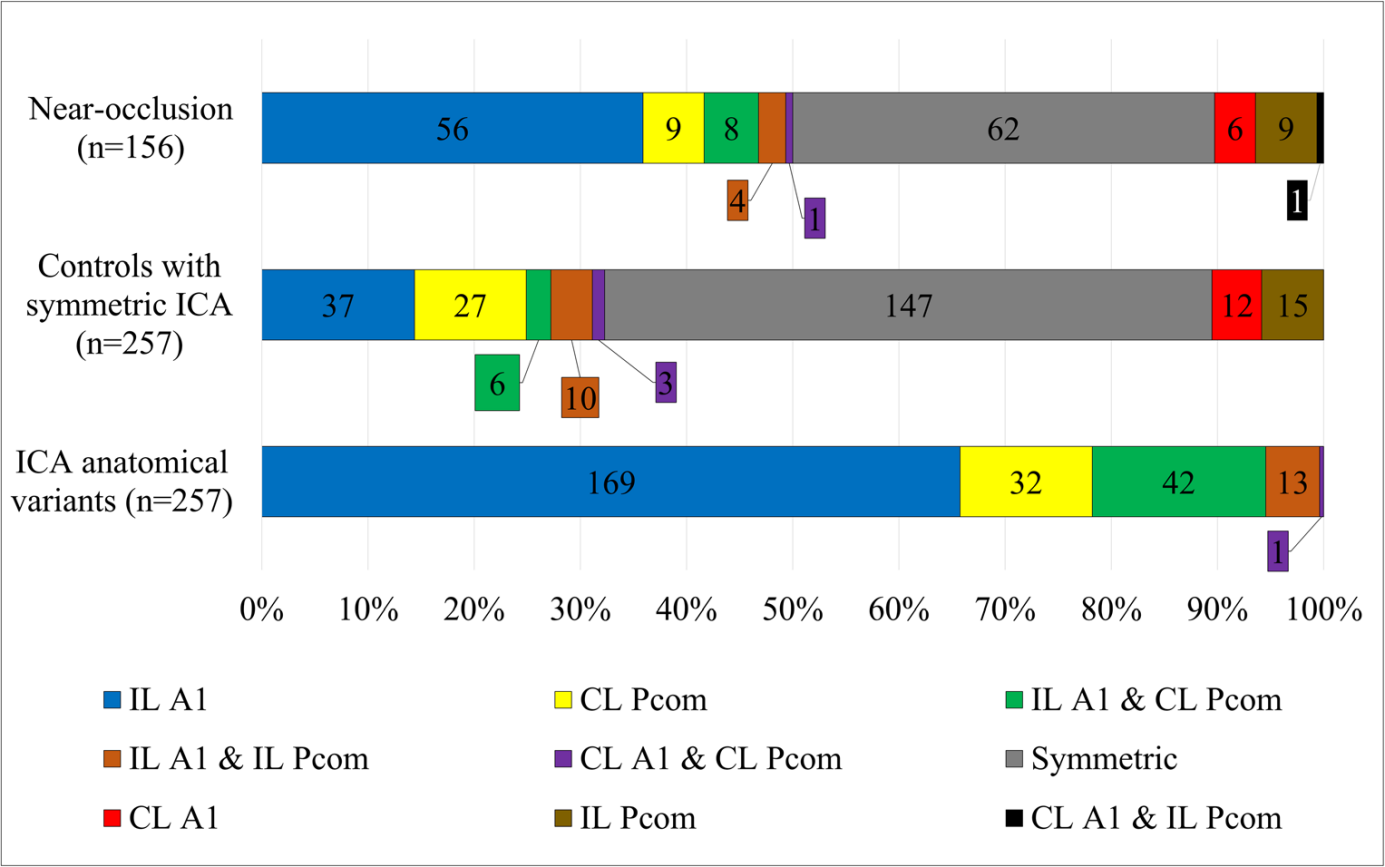
Circle of Willis appearance in ICA anatomical variants and controls with symmetric ICA. A1: Side of smaller A1. ICA: Internal carotid artery. Pcom: Side of larger Pcom. Ipsilateral (IL) and contralateral (CL) denotes side of smaller ICA (in both symmetric and asymmetric cases)

Of the 257 asymmetric cases, 71 (28%) had a ≥ 1.0-mm side-to-side difference in ICA diameter, which was also seen in 93% of near-occlusions. CoW anatomical variants were also noted in 32% of controls with symmetric ICAs and in 50% of near-occlusions (Fig. 2).

## Discussion

CoW anatomical variants associated with ICA asymmetry in more than half of our sample was present in all ages, both sexes, and without steno-occlusive disease. When persons with such ICA asymmetry develop a moderate carotid stenosis, that stenosis will mimic a carotid near-occlusion. In contrast, the hallmark of near-occlusion is a severe stenosis that causes the distal ICA to reduce in size. For this reason, relying on ICA asymmetry alone (such as a ≥ 1.0-mm side-to-side diameter threshold [6]) is insufficient for a near-occlusion diagnosis as we have demonstrated that 28% of ICA anatomical variants without ≥ 50% stenosis had a ≥ 1.0-mm side-to-side diameter difference and because a few near-occlusions had less side-to-side diameter difference.

CoW anatomical variance was also quite common among controls without ICA asymmetry. However, a small ipsilateral A1 seemed to be more strongly associated with ICA asymmetry compared than a large contralateral Pcom (Fig. 2).

In near-occlusion, asymmetric CoW is often noted, presumably caused by pathology (reduced/reversed collateral flow), whereas in CoW anatomical variations, the cause is presumably congenital (hypoplasia/aplasia). Since the mechanism of CoW appearance (pathological versus congenital) is very difficult to determine with CTA, CoW appearance is likely of limited value when distinguishing between these causes if ICA asymmetry; rather, a systematic approach of several extracranial features seems reasonable [1, 8]. Given that ICA size can change with changes in flow [1, 9], the small distal ICA in congenital cases is reasonable by CoW variants; however, it cannot be excluded that a small distal ICA congenitally result in CoW variation, why we described this as an association.

ICA anatomical variance seemed to be more common when coinciding with a stenosis than when not, especially ipsilateral stenosis. This was possibly caused by our conservative estimate of near-occlusions, categorizing some actual near-occlusions as caused by anatomical variance. This is sometimes a difficult determination, with some cases eluding certain diagnosis.

Limitations of this study are non-uniform exam protocols although protocols are reflective of standard clinical practice and therefore generalizable to a broader population. Additional limitation is lack of postoperative imaging that otherwise might improve the certainty in near-occlusion (distal ICA should expand to symmetry) and near-occlusion mimic (distal ICA should remain asymmetric). Visual assessment was used to determine if a case was asymmetric as there is no well-accepted measured threshold for ICA asymmetry; however, the outcome (a ≤ 0.88 ICA ratio) was very similar to a previous study assessing near-occlusion (≤ 0.87 ICA ratio) [8]. A1 and Pcom diameters were not measured.

In summary, ICA asymmetry associated with CoW anatomical variation commonly mimic near-occlusion when coinciding with stenosis. Thus, when determining if carotid stenosis is a near-occlusion (the first step in all NASCET-grading of carotid stenosis), it is important to not rely solely on ICA asymmetry but interpret several features of near-occlusion. It seems disappointing that a measurement or measurements cannot be recommended to solely diagnose near-occlusion [8].
